# Upwelling jet separation in the California Current System

**DOI:** 10.1038/s41598-018-34401-y

**Published:** 2018-10-30

**Authors:** Renato M. Castelao, Hao Luo

**Affiliations:** Department of Marine Sciences, University of Georgia, Athens, GA USA

## Abstract

The California Current System is characterized by summertime wind-driven upwelling, high biological productivity, and an intense equatorward upwelling jet. The upwelling jet is generally located close to shore to the north of Cape Blanco (43°N), but it separates from the coast at the cape during summer extending farther offshore downstream of the separation point. Jet separation results in a wider region influenced by cold, nutrient-rich upwelled waters, strongly affecting biological productivity, mesoscale activity, and air-sea interactions. Flow-topography interactions are thought to play a dominant role in jet separation. Here, we use a high-resolution ocean model to show that the wind stress curl is a dominant forcing controlling jet separation, and that separation can occur independently of flow-topography interactions. While jet separation occurs in simulations with realistic wind stress curl and modified topography with no submarine banks or capes, jet separation is substantially reduced when the wind stress curl is removed, even in the presence of realistic topography. This novel insight indicates that future changes in winds, as the predicted delay in the seasonal development of wind stress curl intensifications, may result in substantial changes in ocean circulation in the California Current System.

## Introduction

Eastern Boundary Current Systems have long been recognized as highly productive zones. In the California Current System off the U.S. West Coast, predominant equatorward winds during summer^1^ result in net offshore transport in the surface Ekman layer, and upwelling of cold, saline, nutrient-rich water near the coast^2^. A strong alongshelf coastal jet that is in geostrophic balance with the upwelled isopycnals is formed^3^. In regions of alongshelf uniform topography (both bottom bathymetry and coastline orientation), the upwelling circulation is generally well described by standard two-dimensional models^4^. In the presence of alongshelf variations in topography, however, highly three-dimensional flow patterns are often observed. During summer, the upwelling jet separates from the coast near Cape Blanco (43°N) to become an oceanic jet to the south (Supplementary Fig. 1)^5^. While upstream of the cape the summer-averaged upwelling jet lies about 15–30 km offshore, it is located farther from the coast to the south of Cape Blanco (>100 km from shore) after separation^5^. The offshore displacement of the upwelling jet results in strong cross-shelf transport and provides an important mechanism for exporting material from the highly productive continental shelf^6^. This results in a broader area influenced by the upwelling circulation to the south of Cape Blanco compared to regions to the north^5,7,8^, with important consequences for both the coastal and adjacent deep-ocean ecosystems.

The dynamics of jet separation at Cape Blanco and at other capes in other Eastern Boundary Current Systems are still not fully understood^9^. Observational^5,10^ and numerical^11,12^ and laboratory^13^ modeling studies have shown that irregularities in coastline and bottom topography are important for jet separation and the formation of meanders and eddies. Variations in vorticity resulting from curvature of the trajectory as the flow passes a cape result in enhanced upwelling in the lee of topography perturbations^14^. Other potential factors include enhanced wind stress in the lee of capes resulting in stronger offshore Ekman transport^15^, wind stress curl intensifications in the lee of capes^12,16^, the combination of alongshore pressure gradients and wind relaxations^17^, and interactions of the upwelling jet with poleward undercurrents^5^. Many of these factors can occur simultaneously, making it difficult to isolate the contribution from the various mechanisms. For example, wind stress and wind stress curl intensifications are often observed in the lee of capes in the California Current System as a result of orographic effects^18^. Despite the recognition of the potential influence of other factors, flow-topography interactions are thought to be of critical importance for jet separation and the development of recurrent filaments that are anchored at specific locations in Eastern Boundary Current System^19,20^. In the Canary Current System, for example, interactions of the flow with the bottom bathymetry have been suggested to be necessary for triggering the formation of a filament anchored at Cape Ghir (30.5°N)^12^. In the absence of coastal protuberances, jet separation and the formation of standing features that extend far offshore off capes are not observed in many laboratory^13^ and numerical^16^ modeling studies, suggesting that they are produced as the result of interaction of the upwelling circulation with the coastal irregularities themselves^13^.

In this study, we use high-resolution ocean model simulations to identify the relative contributions of interactions of the flow with Cape Blanco and of intensifications in wind stress and in wind stress curl that are observed in the lee of the cape on jet separation in the California Current System. We show that the wind stress curl intensification in the lee of the cape is more important than the interactions of the flow with the cape itself to drive jet separation and to enhance the offshore export of recently upwelled, nutrient-rich waters.

## Results

**Figure 1:**
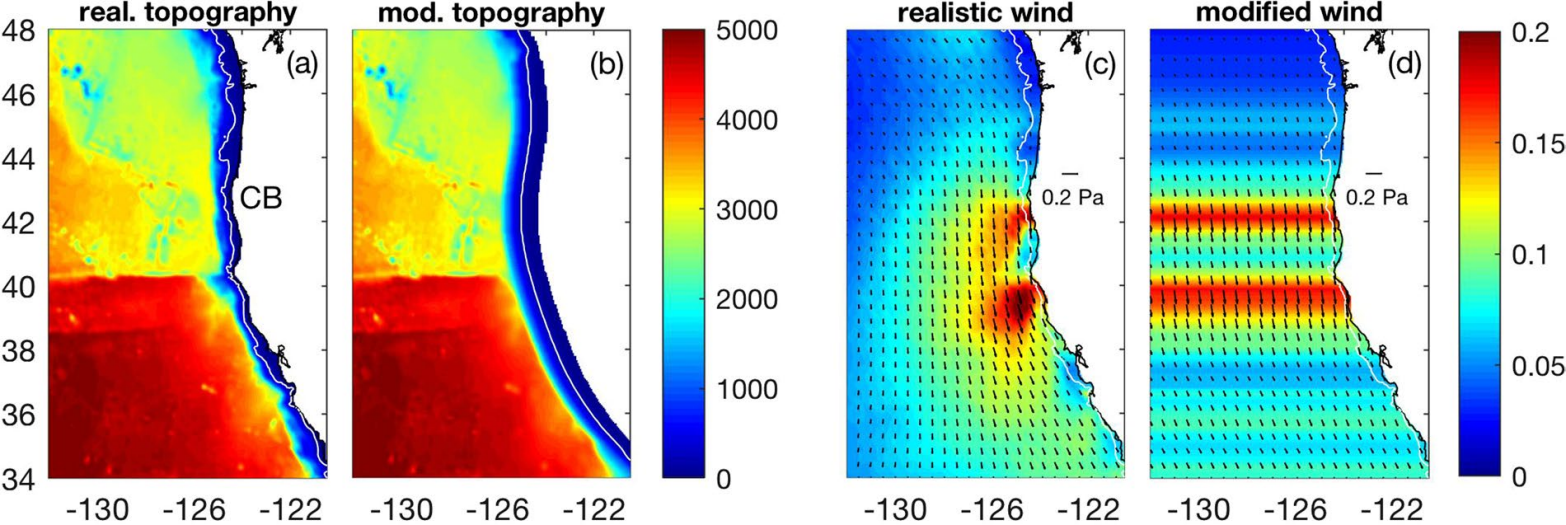
(**a**) Realistic bottom topography and (**b**) modified topography with capes and submarine banks removed (in meters). Average for July of (**c**) realistic and (**d**) modified wind (in Pa), as an example. Wind near the coast is similar in both cases, resulting in similar amounts of wind-driven coastal upwelling. Modified wind is characterized by near-zero wind stress curl. CB: Cape Blanco.

**Figure 2:**
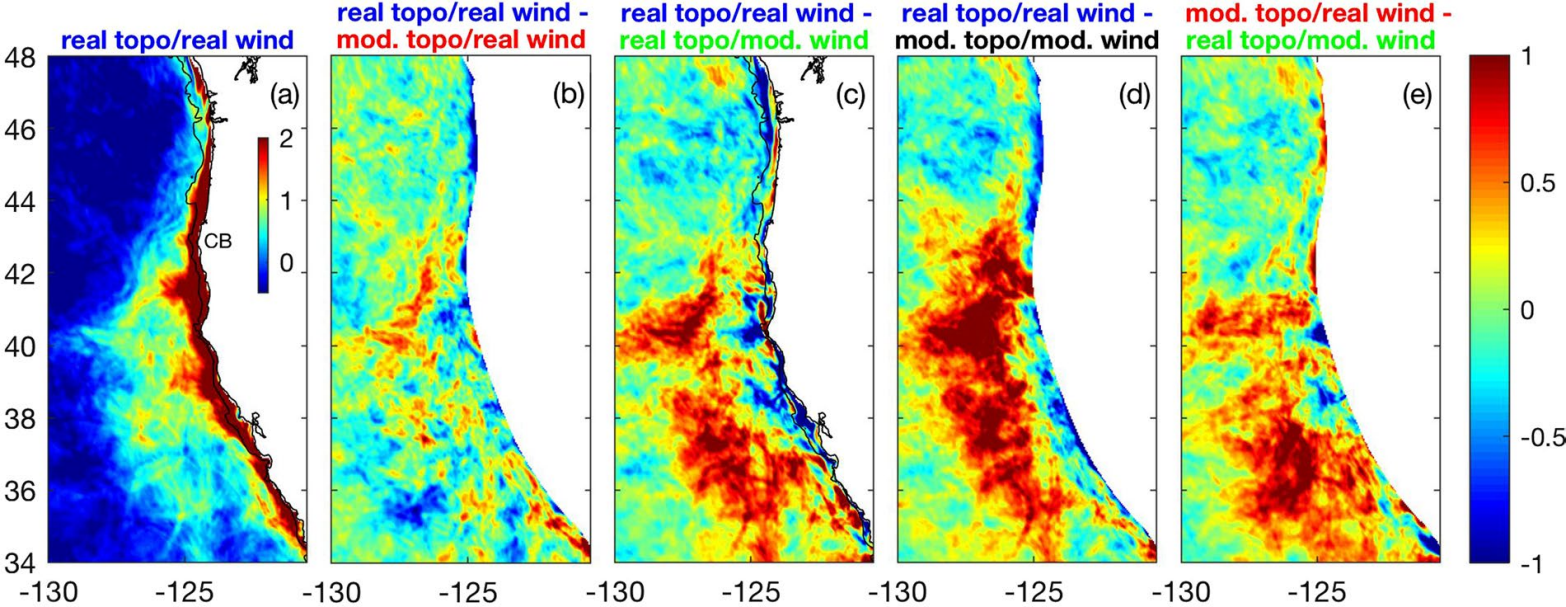
(**a**) Dominant empirical orthogonal function (EOF) of SST gradients (°C per 100 km) in the California Current System for case using realistic topography and realistic wind. Amplitude time series is shown in Supplementary Fig. 2. Remaining panels show difference between EOF of SST gradients for the realistic case (shown in panel a) and for the various idealized scenarios: (**b**) modified topography with no banks/capes and realistic wind, (**c**) realistic topography and modified wind with no curl, (**d**) modified topography and modified wind. (**e**) Difference in dominant EOF between simulation using modified topography with no banks/capes and realistic wind and simulation using realistic topography and modified wind with no curl. Only regions where topography is identical between the two simulations being compared are shown. CB: Cape Blanco.

**Figure 3:**
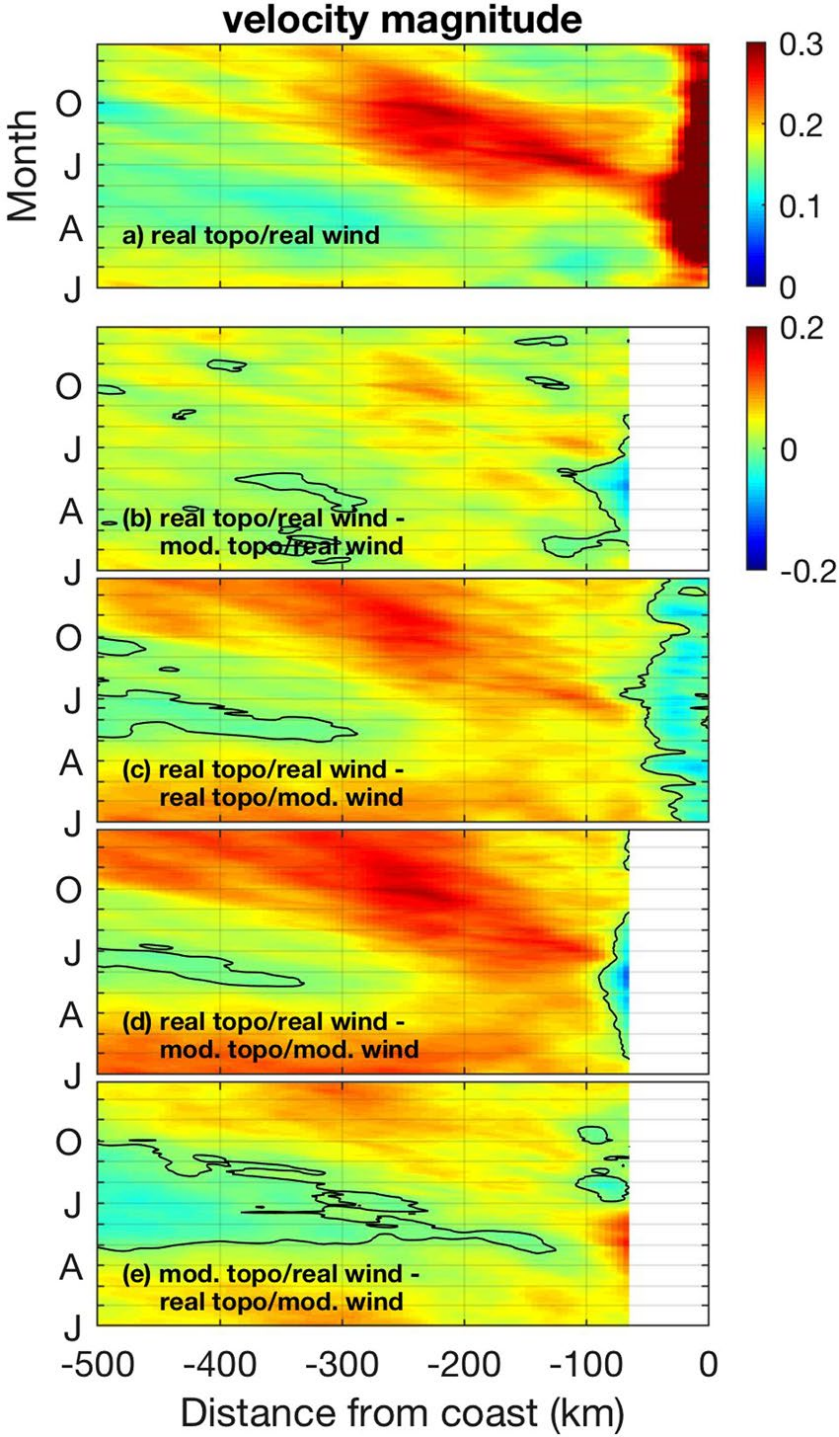
(**a**) Seven-year average of surface velocity magnitude (m s^−1^) averaged between 38°–43°N as a function of distance from coast and time for case using realistic topography and realistic wind. Intensification far from the coast starting in late spring/early summer is due to jet separation. Remaining panels show difference between velocity magnitude for the realistic case (shown in panel a) and for the various idealized scenarios: (**b**) modified topography with no banks/capes and realistic wind, (**c**) realistic topography and modified wind with no curl, (**d**) modified topography and modified wind. (**e**) Difference in surface velocity magnitude between simulation using modified topography with no banks/capes and realistic wind and simulation using realistic topography and modified wind with no curl. Only regions where topography is identical between the two simulations being compared are shown. Zero contour is shown in black.

**Figure 4:**
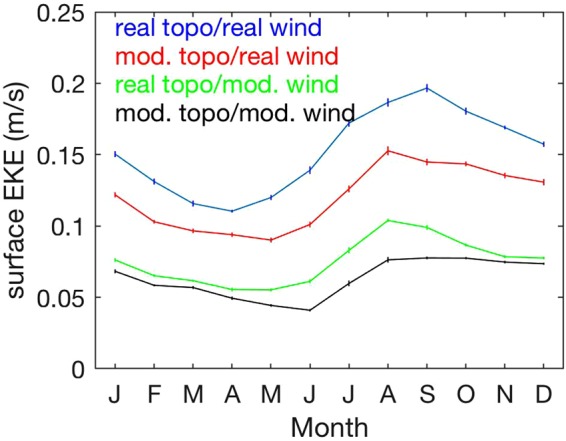
Seven-year average of surface eddy kinetic energy (EKE) cast as a speed, ${({u}^{2}+{v}^{2})}^{1/2}$, where *u* and *v* are the horizontal components of the surface velocity after subtracting the respective monthly averages. Time series are spatial averages for 127.5°–125.5°W, 38°–43°N, color-coded for the different simulations. Error bars represent standard errors of the mean.

In order to identify mechanisms driving jet separation in the California Current System, we consider four different scenarios: (1) realistic topography and realistic wind forcing (Fig. 1a,c), (2) modified topography with no capes or submarine banks and realistic wind forcing (Fig. 1b,c), (3) realistic topography and modified wind with no curl (Fig. 1a,d), and (4) modified topography with no capes/banks and modified wind with no curl (Fig. 1b,d). We examine the evolution of sea surface temperature (SST) gradients away from the coast because SST fronts have been shown to be good proxies for the location of the upwelling jet in the California Current System and in other Eastern Boundary Current Systems^5,21–24^. Consistent with satellite observations^25^, model results during summer using realistic topography and wind forcing (Fig. 1a,c) are characterized by a relatively narrow band of strong SST gradients to the north of Cape Blanco which broadens substantially to the south of the Cape (Fig. 2a, Supplementary Fig. 2a,b). The widening of the region of strong SST gradients to the south of Cape Blanco during summer is tightly coupled to the offshore movement of the upwelling jet (Fig. 3a) as it separates from the coast at the cape^5,26^ (see Supplementary Fig. 1 for an example).

In order to identify the contributions of topography, wind stress and wind stress curl to jet separation and to the widening of the region of strong frontal variability downstream of Cape Blanco, we compare the evolutions of SST gradients and surface velocity magnitudes away from the coast among the various scenarios considered. For the case with realistic wind but with modified topography with no capes or submarine banks (Fig. 1b,c), the seasonal intensifications of both SST gradients (Fig. 2b, Supplementary Fig. 2b,c) and of surface velocities (Fig. 3b) far from shore are only slightly reduced compared to the original scenario in which both topography and winds are realistic. This indicates that, surprisingly, the intensifications of velocity and SST gradients far from the coast that are associated with jet separation occur even in the absence of coastal capes and submarine banks.

Results are substantially different when the wind stress curl is neglected, however, even when capes and submarine banks are considered (Fig. 1a,d). In that case, the offshore region to the south of Cape Blanco between 35° and 43°N is characterized by considerably weaker summer intensification in SST gradients (coherent regions offshore with positive differences in EOF of SST gradient magnitudes to the south of 43°N in Fig. 2c; compare also Supplementary Fig. 2b,d) and by weaker surface currents (Fig. 3c). The region close to shore to the south of Cape Blanco, on the other hand, is characterized by stronger SST gradients and surface currents during summer compared to the basic case considering realistic topography and winds (negative values near the coast in Figs 2c and 3c). This indicates that jet separation is reduced in this scenario of realistic topography and modified wind with no curl, with the upwelling jet and its associated signatures (strong surface currents and SST gradients) remaining closer to the coast. Note that jet separation is reduced even though realistic topography including capes and banks is used and the intensification in the equatorward wind stress within 100 km from the coast in the lee of Cape Blanco is similar to the simulation considering realistic forcing (compare Fig. 1c,d). This suggests that reduced jet separation (Figs 2c and 3c) occurs due to the absence of the wind stress curl intensification in the lee of the cape. When capes and submarine banks are also removed (in addition to the wind stress curl; Fig. 1b,d), jet separation is further reduced resulting in even weaker SST gradients (Fig. 2d, Supplementary Fig. 2e) and surface velocities (Fig. 3d) offshore during summer.

Eddies and other mesoscale activity have been shown to play a critical role on physical and biological processes in the California Current System^23^, influencing the distribution of nutrients^27^, carbon^28^ and SST gradients^29^. Comparing results from the different scenarios reveal substantial differences in eddy kinetic energy – a measure of the intensity of mesoscale activity – that is consistent with the distribution of mean SST gradients and mean surface currents. Eddy kinetic energy offshore is somewhat reduced in the simulation with realistic wind and modified topography with no capes/banks compared to the simulation considering realistic wind and topography, but the decrease is larger in the simulations forced by modified wind with no curl, even in the presence of realistic topography (Fig. 4). This is consistent with jet separation being stronger in the scenario with realistic wind stress curl and modified topography with no capes/banks compared to the scenario with realistic topography and modified wind with no curl (Figs 2e and 3e, Supplementary Fig. 2c,d).

## Discussion

Collectively, our results suggest that the wind stress curl is a dominant factor controlling jet separation in the California Current System, being possibly more important than the direct influence of topographic perturbations. This is surprising, considering the vast literature suggesting that flow-topographic interactions play a dominant role controlling jet separation in Eastern Boundary Current Systems^5,6,11,20,30,31^. Some previous studies have failed to produce jet separation in the absence of topographic perturbations, which is in contrast with our current results (e.g., Fig. 2b; Supplementary Fig. 2c). However, these studies often did not consider a realistic representation of the wind stress curl field^13,30^ or they covered a relatively small area and used boundary conditions that acted to inhibit jet separation^16^. We note also that filament formation due to the finite-amplitude evolution of instabilities associated with the coastal upwelling front do occur in model simulations using a straight coast^32^. This is different than jet separation, however, in the sense that they do not occur repeatedly at a given location, in contrast to jet separation that occurs repeatedly off Cape Blanco^5^ (Fig. 2a; see also Supplementary Fig. 1 for an example). As such, these filaments associated with instabilities^32^ are observed in instantaneous fields but their signatures are smoothed out in seasonal or longer-term averages.

**Figure 5:**
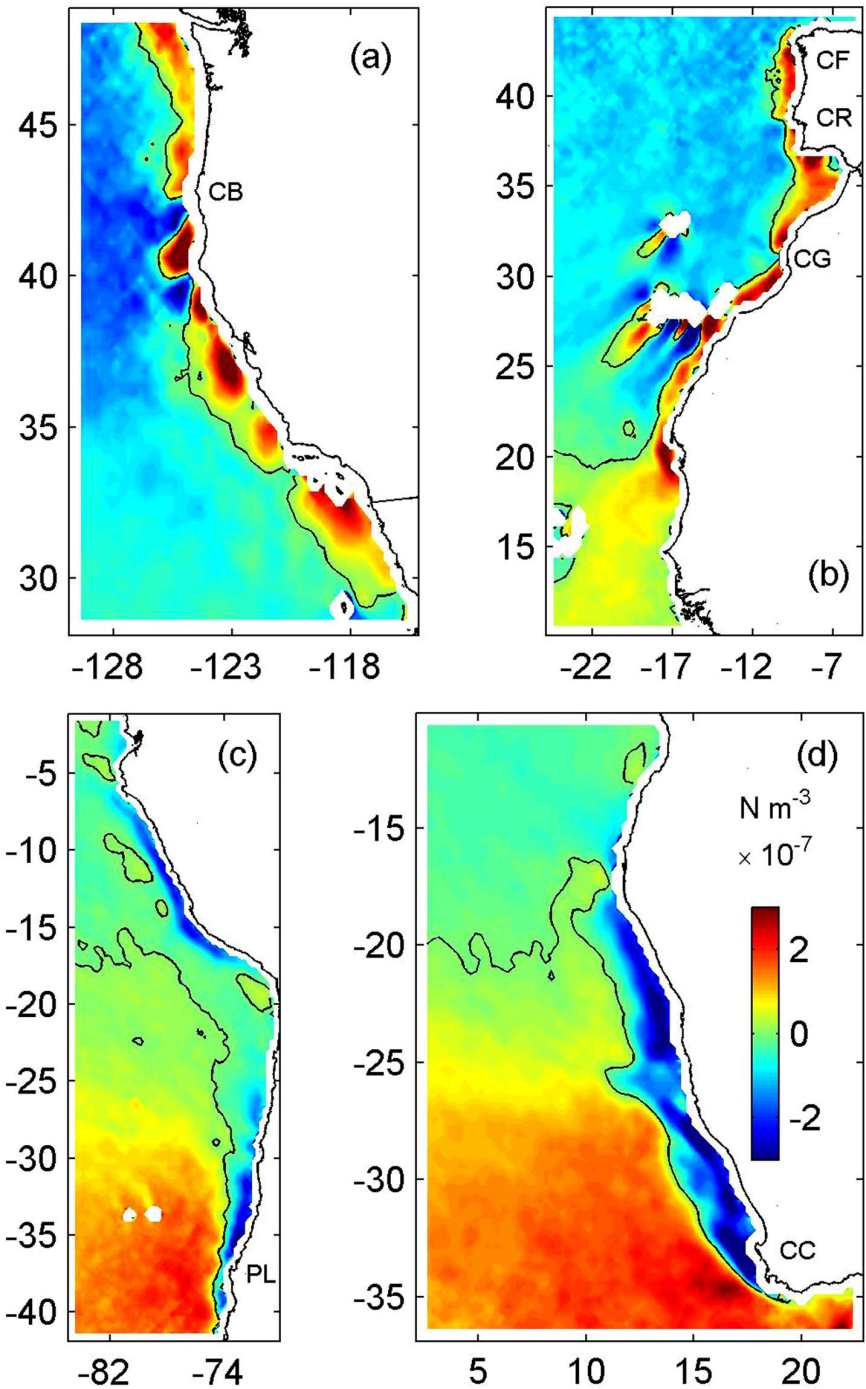
Satellite (QuikSCAT)-derived average summertime wind stress curl (×10^−7^ N m^−3^) in the (**a**) California, (**b**) Canary, (**c**) Humboldt and (**d**) Benguela Current Systems. Positive (negative) wind stress curl intensifications favor upwelling in the northern (southern) hemisphere. Jet separation has been reported to occur equatorward of Cape Blanco (CB), Cape Finisterre (CF), Cabo Roca (CR), Cape Ghir (CG), Punta Lavapie (PL) and Cape Columbine (CC). Note wind stress curl intensifications favoring upwelling in the lee of each of these capes.

It is well established from observations and models that localized wind stress intensifications in the California Current System (and in other Eastern Boundary Current Systems) are often related to orographic effects, such as airflow accelerating within expansion fans that develop downwind of major capes along the California coast^18,33,34^. As such, localized intensifications in wind stress and wind stress curl are generally observed in the lee of capes^15^ (Fig. 5), which makes it difficult to isolate the effects of topography and of wind stress curl intensifications on the separation of upwelling jets based on observational or realistic modeling efforts alone. Our process-oriented study suggests that the direct interaction of the flow with the topography at Cape Blanco can induce some separation of the upwelling jet from the coast and consequently result in the widening of the region with high SST gradients downstream of the separation point (Supplementary Fig. 2d; this is consistent with previous studies^13,16^). However, the most important effect of the cape may be indirect: The presence of the cape results in an orographic intensification of the wind stress in its vicinity^35^, which leads to the establishment of a strong wind stress curl intensification in the lee of the cape^36^. The wind stress curl intensification has been shown to aid jet separation via continuity and by creating potential vorticity contours that track far offshore of the cape^16^, a result supported by later studies^37,38^. Thus, rather than directly influencing separation, our process-oriented study suggests that the main role of Cape Blanco in the California Current System is to modify the wind stress curl field, and the wind stress curl then influences jet separation.

Our results indicate that jet separation at Cape Blanco in the California Current System (and presumably at other capes in other Eastern Boundary Current Systems) is strongly influenced by wind stress curl intensifications in the lee of the cape. Jet separation substantially increases the three-dimensionality of the flow downstream of the separation point^5,8,30^, with the development of an intense meandering flow regime that dominates the circulation in the region^23^. This exerts strong control over ocean-atmosphere interactions^39,40^, eddy^41^ and other meso^23^ and submesoscale activity^42^, and on the cross-shelf export of chemical and biological materials^27^, including the injection of carbon recently fixed though photosynthesis in highly productive coastal regions into the adjacent deep ocean^43^. Therefore, our results suggest that wind stress curl intensifications in the lee of capes play a critical role on these processes. This is especially important in light of recent studies^44^ that support Bakun’s^45^ original hypothesis that increasing greenhouse gas concentrations would force intensification of upwelling favorable winds in Eastern Boundary Current Systems because of increased land-ocean temperature gradients. Model results also predict strong increases in wind stress curl in the northern California Current System^46^ from July through October^47^, and a decrease in intensity from April to June^47^. The strength of wind stress curl intensifications in the lee of capes can influence the timing of jet separation^16^. As such, a delay in the establishment of the wind stress curl intensification in the lee of Cape Blanco (i.e., weaker wind stress curl in spring intensifying later on during summer^46,47^) may result in a delay in jet separation in the California Current System. The anomalous wind conditions observed in the northern California Current System in 2005, when early-season upwelling winds were delayed by 1 month and late-season upwelling winds were stronger than average, substantially altered the nearshore coastal ocean ecosystems resulting in reduced availability of nutrients and smaller densities of recruits of mussels and barnacles in the region^48^. Due to its direct effect on jet separation, future changes in the intensity and seasonality of the wind stress curl field^46,47^ may similarly have a broad influence in the California Current System ecosystem, being particularly important to influence the three-dimensionality of the circulation which ultimately controls the width of the region under the direct influence of nutrient-rich upwelled waters. Knowing whether the wind stress curl plays a similar role in other upwelling systems is important, since jet separation at capes characterized by wind stress curl intensifications in their lees favoring upwelling is a ubiquitous feature in Eastern Boundary Current Systems (Fig. 5). Continued efforts to measure coastal wind stress and wind stress curl fields in high resolution using satellites is also critical to understand how Eastern Boundary Current Systems will respond to future changes in atmospheric forcing.

## Methods

The modeling effort is based on a regional implementation of the Regional Ocean Modeling System (ROMS^49^) to the California Current System. The model has a resolution of 4 km with 30 vertical terrain-following layers from 150° to 115°W, 25° to 48°N, with resolution decreasing progressively to 15 km over the rest of the domain (177°-100°W; 10°-55°N). Vertical mixing is parameterized according to the Large/McWilliams/Doney scheme. Initial and boundary conditions for this regional model are obtained from a ROMS implementation that covers the entire North Pacific Ocean with a resolution of 15 km and 30 vertical layers. The North Pacific Ocean simulation uses initial and boundary conditions from the Simple Ocean Data Assimilation (SODA 2.2.4) reanalysis^50^. The regional model is run from 2000 to 2009, with the first 3 years discarded from analyses as spin up. ROMS is also forced by monthly surface wind stress obtained from the SeaWinds scatterometer onboard NASA’s Quick Scatterometer (QuikSCAT) satellite, and by heat and freshwater fluxes from NCEP North American Regional Reanalysis (NARR). This is essentially the same approach we have recently used to investigate the fate of meltwater runoff from the Greenland Ice Sheet in the Labrador Sea^51^. Surface heat/freshwater fluxes are first averaged zonally in a band extending from 400 to 700 km from shore (i.e., away from the largest upwelling signature) before forcing the model. Tests indicate that this is a necessary step to avoid imposing strong SST gradients in the model due to large variability in heat fluxes in the lee of capes in the California Current System. As such, the surface heat fluxes are imposed as a function of time and latitude only. Surface heat fluxes are employed with nudging toward NOAA extended SST in order to avoid model drifts. NOAA extended SST is first similarly averaged zonally so that large SST gradients are not directly imposed in the model. Even though these procedures result in the imposition of a weak north-south SST gradient, its magnitude is much smaller than the typical SST gradient observed in the California Current System associated with upwelling jets and fronts. As such, SST gradients in the model result almost exclusively from internal dynamics. Although the idealized nature of the heat fluxes used as forcing precludes direct comparisons with observations, the model is able to capture the seasonal evolution of SST gradients in the region, with the dominant empirical orthogonal function (EOF; Supplementary Fig. 2a,b) presenting spatial and temporal characteristics that are consistent with those from the dominant EOF of satellite-derived SST gradients^25^.

Additional idealized model simulations were pursued to isolate the contribution of topography and wind forcing on jet separation in the California Current System. In the modified topography case, capes and submarine banks within approximately 60 km from the coast are eliminated (compare Fig. 1a,b), while offshore of that location the topography remains unaltered. Although the large-scale orientation of the coastline is preserved, the radius of curvature of the shelf topography/coastline is very large (1000’s of km). As such, jet separation is not strongly influenced by flow-topography interactions over the shelf, since vorticity resulting from curvature of the trajectory as the flow passes the perturbation is proportional to *V/R*, where *V* is the flow speed and *R* is the radius of curvature of the streamline^14^ (for large *R* ~ 1000’s of km, the term is small). We refer to this topography as modified topography with no capes/banks. Note that both coastline curvature and topography of the ocean floor are modified simultaneously, so the relative contribution of bottom bathymetry and of capes on jet separation cannot be isolated with the simulations presented here.

In other idealized scenarios, the wind stress is modified in order to remove the wind stress curl. Specifically, QuikSCAT winds are initially zonally averaged within 100 km from the coast. Those values are then kept constant in the zonal direction. As a result, ${\rm{\partial }}{\tau }^{y}/{\rm{\partial }}{\rm{x}}=0$, where ${\tau }^{y}$ is the meridional component of the wind stress and *x* is the zonal direction. For each month, we additionally compute averages of the zonal component of the wind stress (${\tau }^{x})$ within 100 km from the coast for regions between 34°–37°N, 40°–42°N, and 45°–48°N. The zonal component of the wind stress is then linearly interpolated between these 3 latitudinal ranges. As a result, ${\tau }^{x}$ varies smoothly in the alongshore direction *y* and ${\rm{\partial }}{\tau }^{x}/{\rm{\partial }}{\rm{y}}$ is substantially reduced compared to observations, approaching zero. With this procedure, the wind stress curl ${\rm{\partial }}{\tau }^{y}/{\rm{\partial }}{\rm{x}}-{\rm{\partial }}{\tau }^{x}/{\rm{\partial }}{\rm{y}}$ is approximately zero. We refer to this wind stress field as modified wind with no curl. Note that the wind stress within 100 km from the coast in the realistic wind and in the modified wind with no curl cases are similar to each other (compare Fig. 1c,d), resulting in comparable amounts of coastal upwelling. Alongshore convergences and divergences in wind stress within approximately 100 km from the coast are also similar between the two cases, although they are enhanced offshore in the modified wind case. In scenarios forced by realistic topography and modified wind with no curl, jet separation in the vicinity of the cape can be influenced by flow-topography interactions and by the wind intensification in the lee of the cape (i.e., by enhanced coastal upwelling), but not by the wind stress curl.

## Electronic supplementary material

Supplementary Figures

## Data Availability

The data/reanalysis that support the findings of this study are publically available online at, http://podaac.jpl.nasa.gov/QuikSCAT, www.atmos.umd.edu/~ocean/, and www.esrl.noaa.gov/psd/data/gridded/data.narr.html. Model codes are available at, www.myroms.org.
