# Supplementary material for: Upwelling jet separation in the California Current System
Source: Sci Rep. 2018 Oct 30;8:16004. doi: 10.1038/s41598-018-34401-y (PMC6207725; doi:10.1038/s41598-018-34401-y)
Supplement: Supplementary file 1 — Supplementary Figures [file 41598_2018_34401_MOESM1_ESM.pdf]

**< Supplementary Information >**

# Upwelling jet separation in the California Current System

Renato M. Castelao* and Hao Luo

Department of Marine Sciences, University of Georgia, USA

*Corresponding author: castelao@uga.edu

**Supplementary Figures**

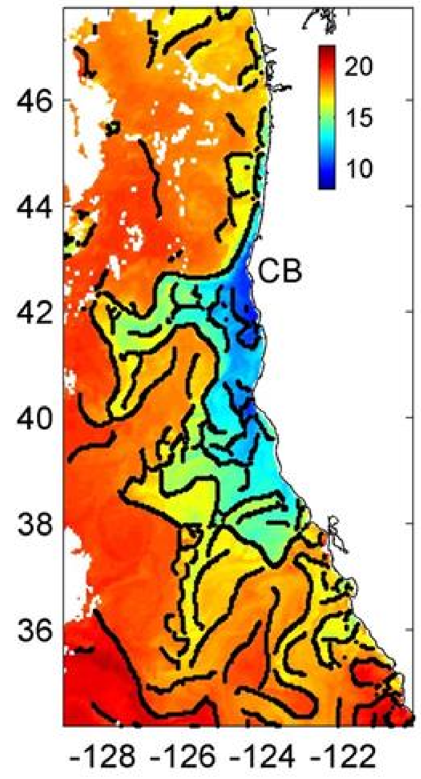


**Supplementary Figure 1 |** Sea surface temperature (°C) in the California Current System on September 5th, 2004. Black lines indicate the location of strong SST gradients, which are closely related to the location of the upwelling jet[5]. Note that strong SST gradients (and the upwelling jet) are located close to the coast to the north of Cape Blanco, but move sharply offshore at the cape as the upwelling jet separates from the coast. Downstream of the cape, cold upwelled waters reach farther from shore compared to regions north of the cape. White areas denote land or clouds. CB: Cape Blanco.

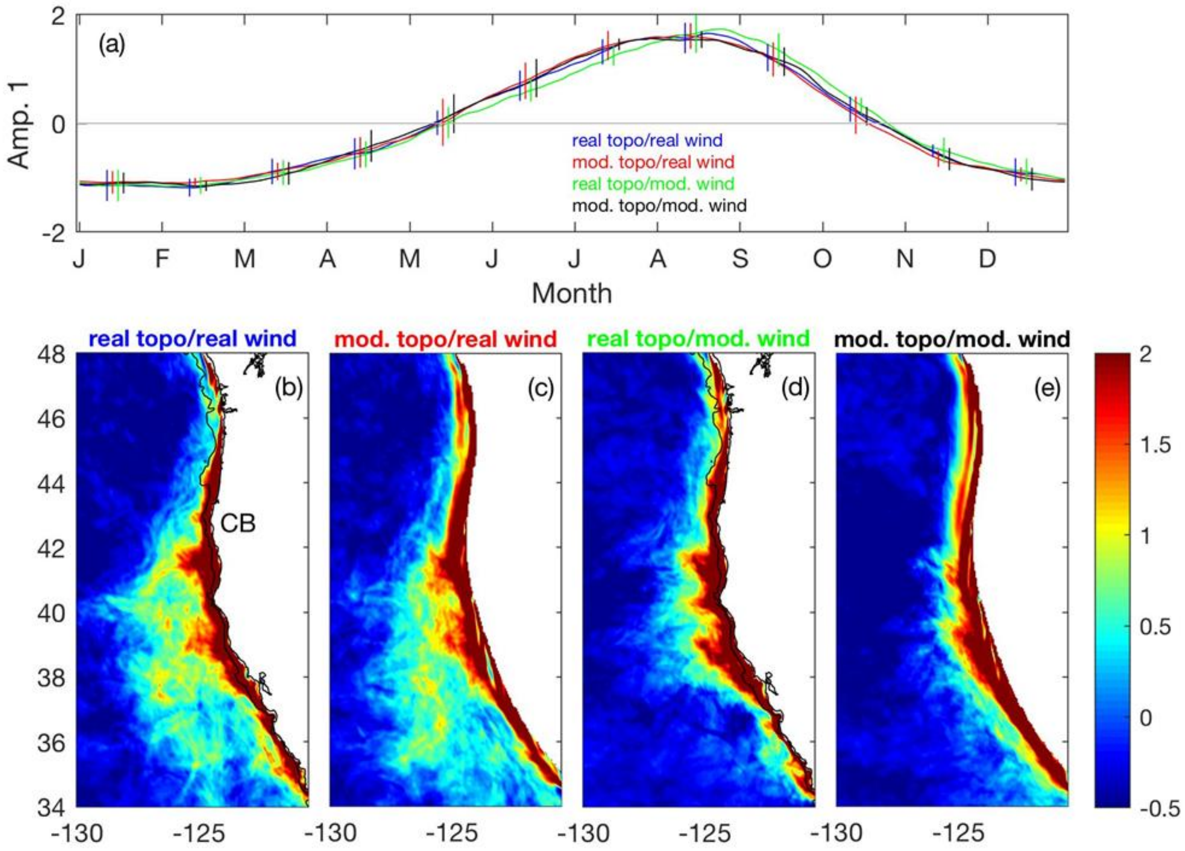


**Supplementary Figure 2 |** EOF decomposition of SST gradients (°C per 100 km) in the California Current System. (a) Seven-year average of amplitude time series for mode 1, color-coded for the different simulations. Error bars represent standard errors of the mean. Spatial pattern of dominant EOF mode for simulation using (b) realistic topography and realistic wind, (c) modified topography with no banks/capes and realistic wind, (d) realistic topography and modified wind with no curl and (e) modified topography and modified wind. CB: Cape Blanco.
